# Study of the irisin hormone on metabolism and mitochondrial dynamics in experimental models of Alzheimer’s disease

**DOI:** 10.1002/alz.091085

**Published:** 2025-01-03

**Authors:** Ariene Soares Fonseca, Mariana Gomes Chauvet, Sergio T Ferreira, Mychael V Lourenco

**Affiliations:** ^1^ Federal University of Rio de Janeiro, Rio de Janeiro, Rio de Janeiro Brazil; ^2^ Institute of Biophysics, Federal University of Rio de Janeiro, Rio de Janeiro Brazil; ^3^ Institute of Medical Biochemistry Leopoldo de Meis, Federal University of Rio de Janeiro, Rio De Janeiro, Rio de Janeiro Brazil

## Abstract

**Background:**

Age‐related decrease glucose utilization, coupled with insulin resistance, are key features of AD, resulting in reduced glucose utilization/catabolism and oxidative stress generation. Irisin, an exercise‐induced hormone promoting mitochondrial biogenesis in adipocytes via PGC‐1α, stimulates thermogenic pathways, increases energy expenditure and induces browning of adipose tissue. Further, irisin expression was shown to trigger neuroprotection in AD models.

**Method:**

Here, we assessed whether irisin could prevent potential metabolic deficits induced by soluble Aβ oligomers (AβOs) in hippocampal slices and in C57BL/6 mice.

**Result:**

Our results indicate that irisin produced only subtle changes in the metabolic profile without clearly altering the protein content associated with mitochondrial dynamics in the AβOs‐exposed hippocampus.

**Conclusion:**

Our data suggest that other mechanisms may possibly contribute to the neuroprotective effects of irisin in AD models.

